# Supplementary material for: From sequence to enzyme mechanism using multi-label machine learning
Source: BMC Bioinformatics. 2014 May 19;15:150. doi: 10.1186/1471-2105-15-150 (PMC4229970; doi:10.1186/1471-2105-15-150)
Supplement: Additional file 2 — Java code of ml2db. Additional file ml2db_code.tar.gz contains the Java source code to run the multi-label machine learning experiments and save the results to database. The code’s Javadoc is included. [file 1471-2105-15-150-S2.zip › additional file 2/ml2db/utils/doc/uk/ac/standrews/utils/main/diff/Diff.html]

Diff


JavaScript is disabled on your browser.


- Overview
- Package
- Class
- Use
- Tree
- Deprecated
- Index
- Help

- Prev Class
- Next Class

- Frames
- No Frames

- All Classes

- Summary:
- Nested |
- Field |
- Constr |
- Method

- Detail:
- Field |
- Constr |
- Method


uk.ac.standrews.utils.main.diff

## Class Diff

- java.lang.Object
- - uk.ac.standrews.utils.main.diff.Diff

- ---

    

  ```
  public class Diff
  extends java.lang.Object
  ```

  Compares two collections, returning a list of the additions, changes, and
  deletions between them. A `Comparator` may be passed as an
  argument to the constructor, and will thus be used. If not provided, the
  initial value in the `a` ("from") collection will be looked at to
  see if it supports the `Comparable` interface. If so, its
  `equals` and `compareTo` methods will be invoked on the
  instances in the "from" and "to" collections; otherwise, for speed, hash
  codes from the objects will be used instead for comparison.

  The file FileDiff.java shows an example usage of this class, in an
  application similar to the Unix "diff" program.

- - ### Constructor Summary

    Constructors

    Constructor and Description

    `Diff(java.util.Collection a,
    java.util.Collection b)`

    Constructs the Diff object for the two collections, using the default
    comparison mechanism between the objects, such as `equals` and
    `compareTo`.

    `Diff(java.util.Collection a,
    java.util.Collection b,
    java.util.Comparator comp)`

    Constructs the Diff object for the two collections, using the given
    comparator.

    `Diff(java.lang.Object[] a,
    java.lang.Object[] b)`

    Constructs the Diff object for the two arrays, using the default
    comparison mechanism between the objects, such as `equals` and
    `compareTo`.

    `Diff(java.lang.Object[] a,
    java.lang.Object[] b,
    java.util.Comparator comp)`

    Constructs the Diff object for the two arrays, using the given
    comparator.
  - ### Method Summary

    Methods

    | Modifier and Type | Method and Description |
    | --- | --- |
    | `java.util.List` | `diff()` Runs diff and returns the results. |
    | `java.lang.Integer[]` | `getLongestCommonSubsequences()` Returns an array of the longest common subsequences. |

    - ### Methods inherited from class java.lang.Object

      `equals, getClass, hashCode, notify, notifyAll, toString, wait, wait, wait`

- - ### Constructor Detail


    - #### Diff

      ```
      public Diff(java.util.Collection a,
          java.util.Collection b)
      ```

      Constructs the Diff object for the two collections, using the default
      comparison mechanism between the objects, such as `equals` and
      `compareTo`.


    - #### Diff

      ```
      public Diff(java.util.Collection a,
          java.util.Collection b,
          java.util.Comparator comp)
      ```

      Constructs the Diff object for the two collections, using the given
      comparator.


    - #### Diff

      ```
      public Diff(java.lang.Object[] a,
          java.lang.Object[] b)
      ```

      Constructs the Diff object for the two arrays, using the default
      comparison mechanism between the objects, such as `equals` and
      `compareTo`.


    - #### Diff

      ```
      public Diff(java.lang.Object[] a,
          java.lang.Object[] b,
          java.util.Comparator comp)
      ```

      Constructs the Diff object for the two arrays, using the given
      comparator.
  - ### Method Detail


    - #### diff

      ```
      public java.util.List diff()
      ```

      Runs diff and returns the results.


    - #### getLongestCommonSubsequences

      ```
      public java.lang.Integer[] getLongestCommonSubsequences()
      ```

      Returns an array of the longest common subsequences.


- Overview
- Package
- Class
- Use
- Tree
- Deprecated
- Index
- Help

- Prev Class
- Next Class

- Frames
- No Frames

- All Classes

- Summary:
- Nested |
- Field |
- Constr |
- Method

- Detail:
- Field |
- Constr |
- Method
